# Skeletal Site-specific Effects of Zoledronate on *in vivo* Bone Remodeling and *in vitro* BMSCs Osteogenic Activity

**DOI:** 10.1038/srep36129

**Published:** 2017-01-31

**Authors:** Xue Gong, Wanlu Yu, Hang Zhao, Jiansheng Su, Qing Sheng

**Affiliations:** 1 Department of Prosthodontics, School of Stomatology, Tongji University, Shanghai Engineering Research Center of Tooth Restoration and Regeneration, Shanghai 200072, China

## Abstract

Bisphosphonate-related osteonecrosis of the jaw (BRONJ) has been associated with long-term oral or intravenous administration of nitrogen-containing bisphosphonates (BPs). However, the pathogenesis of BRONJ remains unknown, and definitively effective treatment has not yet been established. Bisphosphonate-related osteonecrosis (BRON) tends to occur in maxillofacial bones. Why this occurs is still unclear. Here we show that zoledronate (Zol) treatment suppresses alveolar bone remodeling after tooth typical clinical and radiographic hallmarks of the human BRONJ, whereas enhances peripheral bone quantity in bone remodeling following injury in the same individuals, shown as increased cortical bone thickness, increased trabecular bone formation and accelerated bone defect repair. We find that the RANKL/OPG ratio and Wnt-3a expression are suppressed at the extracted alveolar sites in Zol-treated rats compared with those at the injured sites of peripheral bones. We also show that Zol-treated bone marrow stromal cell (BMSCs) derived from jaw and peripheral bones exhibit differences in cell proliferation, alkaline phosphatase (ALP) activity, expression of osteogenic and chondrogenic related marker genes, and *in vivo* bone formation capacity. Hopefully, this study will help us better understand the pathogenesis of BRONJ, and deepen the theoretical research.

Bisphosphonates (BPs) represent a major class of bone resorption inhibitors prescribed for the management of skeletal complications such as osteoporosis, Paget’s disease, multiple myeloma, and complications of metastatic malignancy^1^,^2^,^3^. Bisphosphonate (BP) therapy in these patients effectively improves bone mineral density and bone strength, reduces bone pain and incidence of pathological fracture, and dramatically improves the quality of life. Despite these benefits, bisphosphonate-related osteonecrosis of the jaw (BRONJ) has emerged as a devastating side effect induced by long-term BP use, an entity first described in 2003^4^. BRONJ is defined clinically as exposed necrotic bone in the maxillofacial region for a period that exceeds eight weeks in patients with current or previous treatment with BP, without a history of maxillofacial radiotherapy^5^. Patients with BRONJ present various clinical symptoms including soft tissue swelling or ulceration, pain, infection, and exposed necrotic bone in certain severe cases that significantly impairs the quality of life^6^. In the great majority of cases, BRONJ occurs in patients undergoing invasive dental procedures, primarily tooth extraction^7^,^8^, and is usually refractory to conventional treatment modalities such as surgical debridement, antimicrobial therapy and hyperbaric oxygen therapy^9^,^10^,^11^. To date, no one etiological mechanism has been manifested to be specially correlated with the pathogenic process involved in BRONJ development. Therefore, understanding the pathogenesis of BRONJ and sequentially developing an effective modality to prevent and treat this disease have become an urgent issue for patients using BPs.

Various hypotheses surrounding the etiology of BRONJ have been proposed including oversuppression of osteoclastic bone resorption and bone turnover, suppression of angiogenesis, oral bacterial infection, and oral epithelium toxicity. Most scholars^12^,^13^,^14^ believe that BPs directly inhibit osteoclast-mediated bone resorption, ultimately resulting in the oversuppression of bone turnover and the failure of bone remodeling after tooth extraction. In addition, BPs could also inhibit the endothelial cell function^15^,^16^, resulting in the diminished vascularity and reduced blood supply that may contribute to the development of osteonecrosis at bone-remodeling sites. However, these two hypotheses could not explain why bisphosphonate-related osteonecrosis (BRON) reported seems to particularly affect the jaw. Oral bacterial infection and oral epithelium toxicity have thus been implicated as significant pathogenic factors accounting for this phenomenon. Some researchers believe that BPs accumulate in bone tissue at sufficient concentrations to exert direct toxic effects on oral fibroblasts and epithelial cells^17^,^18^,^19^,^20^, which clearly results in delayed soft-tissue healing at bone-remodeling sites leading to secondary infection and necrosis of the underlying bone. However, in some BRONJ cases, osteonecrosis of the jaw has emerged prior to soft tissue injury and infection^6^. It is still a matter of debate whether infection play the primary role in the pathogenesis or merely represent secondary injury^21^,^22^,^23^.

BRON primarily occurs in maxillofacial bones, suggesting that it favors maxillofacial bones over peripheral bones. Why this occurs is still unclear. The skeletal site-specific osteogenic properties of BMSCs could be implicated as the etiology of this site-specific osseous pathology. Developmentally, maxillofacial bones originate exclusively from neuroectoderm and are formed primarily by intramembranous ossification^24^,^25^, whereas peripheral bones develop from mesoderm and undergo both endochondral and intramembranous ossification^26^. Bones formed by two cell types are identical in structure, yet functional differences in bone turnover and mechanical properties at different anatomic sites may exist. Studies have shown that cell differentiation and function of BMSCs derived from jaw and peripheral bones is different^27^. However, our knowledge about proliferative capacity and osteogenic potential of BMSCs or osteoblast precursors after exposure to BPs is mostly obtained from experimental models with cells from peripheral bones. Little is known about differences in cellular and particularly molecular basis for jaw bone BMSCs vs. peripheral bone BMSCs, and effects of BPs on bone remodeling of jaw and peripheral bones are not yet well understood.

Since jaw bone BMSCs are developmentally and phenotypically different from peripheral bone BMSCs, we have hypothesized that BPs modulates cell function and osteogenic potential of BMSCs in a skeletal site-specific pattern. In this study, zoledronate (Zol), a third-generation BP, frequently linked to osteonecrosis of jaw, was used as a model compound to study effects of BPs. We investigated effects of Zol on *in vivo* bone remodeling of jaw and peripheral bones (ilium and tibia) following injury in a rat model; tested proliferative capacity, osteogenic and chondrogenic potential of Zol-treated BMSCs derived from these three distinct skeletal sites; compared *in vivo* bone formation capacity of Zol-treated jaw BMSCs vs. iliac BMSCs in a nude mouse model; determined the expression of osteogenic related cytokines involved in RANKL/RANK/OPG and Wnt signaling pathways. Hopefully, this study will help to elucidate the pathophysiology of BRONJ, and ultimately develop effective approaches for BRONJ treatment to improve the safety of the current widespread use of BP therapy.

## Results

### BP treatment leads to the BRONJ-like disease in rats

**Figure 1:**
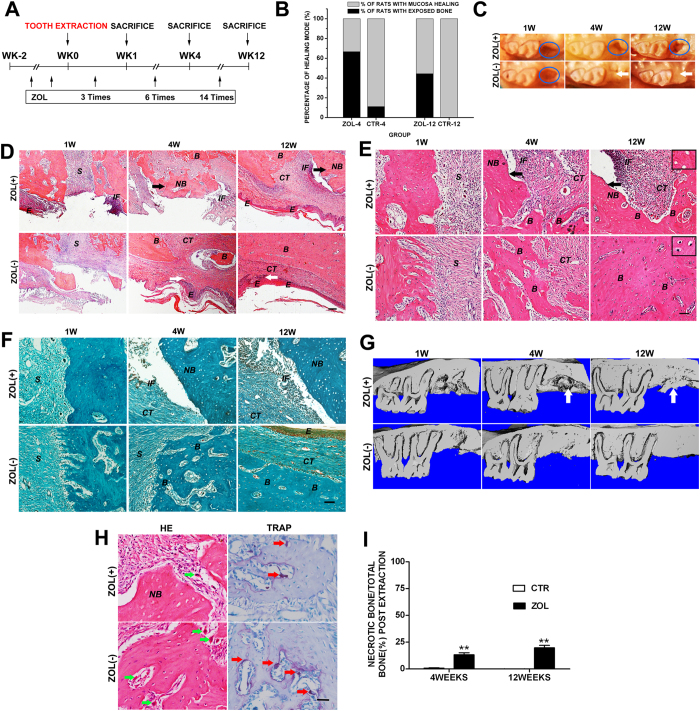
Development of BRONJ-like disease in Sprague-Dawley rats. (**A**) Experimental procedure of inducing a BRONJ-like rat model. Rats were treated intravenously with Zol (80 μg/kg) once weekly for two weeks before extraction of maxillary or mandibular first molars, and the drug was continuously administered once weekly for 1, 4 or 12 weeks. (**B**) Incidence of BRONJ-like lesion, shown as an unhealed open socket with exposed bone at the extraction site in SD rats treated with Zol for 4 and 12 weeks, whereby rats with no Zol treatment served as controls. (**C**) Representative gross clinical appearance of mucosa at the extraction site at 4 and 12 weeks after tooth extraction. Blue circles represent images of the apparent mucosal disruption with exposed bone at the extraction site, whereas white arrows point to the healed mucosa. (**D**) Low magnification images of HE staining of extraction sockets exhibiting impaired mucosal healing with exposed bone and healed mucosa with complete epithelial coverage (white arrows). Black arrows point to the necrotic bone (NB). Scale bar = 200 μm. (**E**,**F**) High magnification images of HE (**E**) and Masson’s trichrome (**F**) staining of extraction sites exhibiting newly formed bone (**B**), necrotic bone (NB), inflammatory infiltration (IF), epithelium (**E**), and connective tissues (CT). Black arrows point to the necrotic bone (NB). Scale bar = 100 μm. The upper right blocks (Scale bars = 50 μm) show the presence of necrotic bone with empty osteocytic lacunae at the extraction sites after 12 weeks of tooth extraction in Zol-treated rats, but show normal osteocytic lacunae in control rats. (**G**) μCT analysis showing necrotic bone (white arrows) at the extracted alveolar socket in Zol-treated rats. (**H**) High magnification images of HE (green arrows) and TRAP staining (red arrows) of extraction sockets exhibiting multinucleate osteoclasts. Scale bar = 50 μm. (**H)** Quantification of bone necrosis in Zol-treated rats at 4 and 12 weeks after tooth extraction. *p < 0.05, **p < 0.01.

The majority of reported BRONJ cases have been correlated with high dose of intravenous nitrogen-containing BPs, primarily Zol and pamidronate (PA)^28^,^29^,^30^, in patients being treated for malignant disease undergoing invasive dental or oral surgery. In this study, we established a rat model of BRONJ-like disease by intravenous administration of Zol following tooth extraction. Sprague-Dawley rats were treated intravenously with Zol at 80 μg/kg of body weight two weeks before extraction of maxillary or mandibular first molars, and the drug was continuously administered weekly until selected time points (Fig. 1A). Untreated rats with the same dental procedures were used as controls. Thereafter, the specimens were retrieved for gross, histological and radiographic observations.

One week after tooth removal, clinical manifestations revealed open sockets with no mucosal coverage in both Zol-treated and untreated control rats (Fig. 1C). Histological observations manifested that the empty alveolar socket in Zol-treated rats was filled with fibroblasts, producing more collagen than that of control rats (Fig. 1E,F). Moreover, an increase in osteoblast number was observed at the peripheral border of the socket in Zol-treated rats. Four weeks after tooth extraction, as shown by clinical observations, 67% (6/9) of Zol-treated rats exhibited impaired mucosal healing and presence of open sockets with exposed bone versus 11% (1/9) in untreated rats (Fig. 1B,C), which was further confirmed by histologic observation (see Table 1). Necrotic bone, identified by the presence of empty osteocytic lacunae, has emerged at the extraction sites in Zol-treated rats (Fig. 1D–F). In untreated rats, the majority of collagen fibers within the extracted sockets were replaced with viable woven bone, suggesting a normal healing course (Fig. 1D–F). Unlike the healing socket of the control rats, the necrotic zone of the alveolar bone in Zol-treated rats harbored fewer multinucleate osteoclasts, based on both HE and TRAP staining after four weeks of tooth extraction (Fig. 1H), indicating that ZOL injection reduced the number of osteoclasts at the extracted alveolar bone. 12 weeks after extraction, as shown by clinical observations, 100% (9/9) of untreated rats healed with complete mucosal coverage, whereas 44% (4/9) of Zol-treated rats failed to heal, revealing a lack of mucosal coverage and presence of bony sequestra at the extraction sites (Fig. 1B,C; Table 1). Histological observations showed major clinical and histological manifestations of the human BRONJ in Zol-treated rats, including an open socket without mucosal coverage, necrotic bone or sequestra with empty osteocytic lacunae, focal osteosclerosis, and inflammatory infiltration (Fig. 1D–F). Clinically, a decrease in the incidence of open sockets, from 67% (6/9) at 4 weeks to 44% (4/9) at 12 weeks, was observed in Zol-treated rats (Fig. 1B,C; Table 1), but the osteonecrosis revealed an increased tendency histologically, shown as enlarged necrotic bone area and increased inflammatory cell infiltration (Fig. 1D–F). However, the viable woven bone was completely replaced with lamellar bone at the extraction sites in untreated rats, manifested as normal wound reepithelialization as well as bone remodeling (Fig. 1D–F). Radiographic evaluation by micro-computed tomography (μCT) showed the presence of a poorly defined alveolar ridge due to impaired bone healing and a mottled trabecular pattern with radiopaque alveolar bone at the extracted alveolar socket in Zol-treated rats (Fig. 1G). These radiopaque bone islands appeared to correlate with the necrotic bone or sequestra exposed in the open sockets. However, the untreated rats revealed remarkable bone filling at the extracted socket, manifested as normal bone regeneration. The extent of osteonecrosis was quantified as the percentage of necrotic bone area over total new bone area. The percentage of necrotic bone in Zol-treated rats was significantly higher than that observed in control rats, and no obvious necrotic bone was observed in control rats. Consistent with an increased tendency to osteonecrosis observed in histological images, the percentage rose from 13.2% at 4 weeks to 19.7% at 12 weeks by quantification of bone necrosis (Fig. 1I).

The above experimental evidences show that administration of Zol leads to the development of BRONJ-like disease in rats undergoing tooth extraction, suggesting that cumulative exposure to BPs is the etiological cause of BRONJ-like disease.

### Skeletal site-specific effects of Zol on bone remodeling in rats

Recent epidemiologic studies show, quite surprisingly, BRON tends to occur in the jaw bone, and the peripheral bone is almost unaffected. Here we examine the effect of Zol on bone remodeling of jaw and peripheral bones following injury in rats, and explore whether BRONJ is jaw-specific. Two weeks after Zol administration, maxillary or mandibular first molar was extracted, and drill hole defect was created on the ipsilateral ilium and tibia in the same individuals. The drug was continuously administered until selected time points for further histological and radiographic analysis.

**Figure 2:**
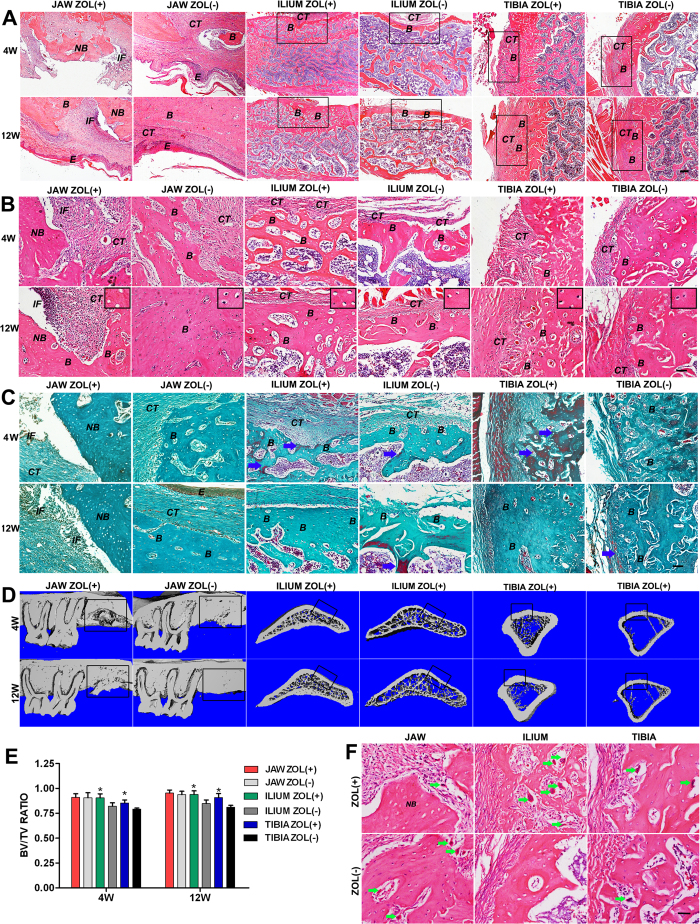
Skeletal site-specific effects of Zol on bone remodeling of jaw, ilium and tibia following injury in a rat model. (**A**) Low magnification images of HE staining at the surgical sites at 4 and 12 weeks following injury. Scale bar = 200 μm. For Zol-treated rats, necrotic bone (NB) developed at the extracted alveolar sites, whereas iliac and tibial bones underwent normal bone remodeling at the surgical sites (black box). (**B**,**C**) High magnification images of HE (**B**) and MT (**C**) staining at the surgical sites at 4 and 12 weeks following injury. Scale bar = 100 μm. The extracted alveolar sites exhibited newly formed bone (**B**), necrotic bone (NB), inflammatory infiltration (IF), and connective tissues (CT) in Zol-treated rats, whereas the surgical sites of peripheral bones showed normal wound healing and bone remodeling, manifested as newly formed bone (**B**) and connective tissues (CT). The upper right blocks (Scale bars = 50 μm) show the presence of necrotic bone with empty osteocytic lacunae at the extraction sites after 12 weeks of tooth extraction in Zol-treated rats, but show normal osteocytic lacunae in other groups. Blue arrows represent incompletely mineralized new bone-like tissues at the surgical sites. (**D**) Three-dimensional reconstruction of μCT images of jaw, ilium and tibia following injury. μCT findings showed necrotic bone or sequestra (white arrows) at the extracted alveolar socket in Zol-treated rats. While for peripheral bones, μCT analysis showed increased cortical bone thickness and trabecular bone volume in the peripheral bones of Zol-treated rats, not only at the defect sites but also at the distal sites. (**E**) Bone histomorphometric analysis. The BV/TV ratio was significantly higher in the peripheral bones of Zol-treated rats but not in the alveolar bone when compared with their respective control rats. (**F**) High magnification images of HE staining exhibiting multinucleate osteoclasts (green arrows) at the surgical sites at 4 weeks following injury. Scale bar = 50 μm.

The above results demonstrate that Zol treatment strongly suppresses alveolar bone remodeling undergoing tooth extraction, and cause BRONJ-like disease in rats that faithfully recapitulates major histological and radiographic features of the human BRONJ based on the following criteria: (1) the presence of an open socket with impaired mucosal healing; (2) the presence of necrotic bone or sequestra with loss of osteocytes from their lacunae; (3) the presence of irregular trabeculae with radiopaque bone that lacked normal bone appearance and matrix structure; (4) the presence of moderate inflammatory infiltration. However, untreated rats undergo normal socket healing, shown as complete epithelial lining and physiological bone remodeling. For peripheral bones, histological analysis showed that all the defects (9/9) healed well in Zol-treated rats within 4 weeks after surgery, and the healed would was surrounded by proliferating fibrous connective tissue (Fig. 2A; Table 1). Furthermore, a significant increase in cortical bone thickness and trabecular bone formation was observed at the injury sites of iliac and tibial bones in Zol-treated rats when compared with their respective control rats (Fig. 2B,C). Additionally, scattered new bone-like tissue (Fig. 2C, blue arrow) was observed at the injury sites of iliac and tibial bones in Zol-treated rats, but very few were present in control rats, indicating that bone formation was more active in Zol-treated rats than that of control rats. 12 weeks after surgery, the injury sites of iliac and tibial bones in Zol-treated rats showed a smooth surface on which the cortex was continuous, and the cortex was thicker and denser than that of their corresponding control rats. Bone remodeling has basically completed at the defect regions in experimental iliac and tibial bones, whereas some new bone-like tissue (Fig. 2C, blue arrow) remained visible in control rats, suggesting that Zol accelerates peripheral bone remodeling. No necrotic lesion in the peripheral bones was observed in both Zol-treated and untreated control rats, and the structure of newly formed bone was also well organized. Interestingly, a marked promotion of bone quantity occurred not only at the defect sites but also at the distal sites of peripheral bones in Zol-treated rats, confirming well-established antiresorptive potency of BPs. μCT findings showed a significant increase in cortical bone thickness and trabecular bone volume in peripheral bones of the Zol-treated rats when compared with untreated rats (Fig. 2D), but showed the presence of irregular trabeculae with radiopaque areas of bone necrosis in the intrabony alveolar sockets of Zol-treated rats (Fig. 2D), which is consistent with histological result described above. Bone histomorphometric analysis demonstrated a significant increase in bone volume fraction (bone volume/total volume, BV/TV) in peripheral bones of the Zol-treated rats but not in the alveolar bone when compared with their respective control rats (Fig. 2E). Consistent with the higher bone remodeling activity at the injury sites of peripheral bones in Zol-treated rats, numerous multinucleate osteoclasts (Fig. 2F, green arrows) were observed in peripheral bones of the Zol-treated rats at 4 weeks after surgery, but fewer and smaller multinucleated cells were found in control rats, as shown by high-magnification HE images. Instead, the viable lamellar bones taken from the extraction sockets of untreated samples contained a large number of multinucleate osteoclasts (Fig. 2F, green arrows), whereas the necrotic zone of the Zol-treated rats was largely devoid of multinucleate cells.

### Disparate effects of Zol on cytokine expression of jaw and peripheral bones following injury in rats

**Figure 3:**
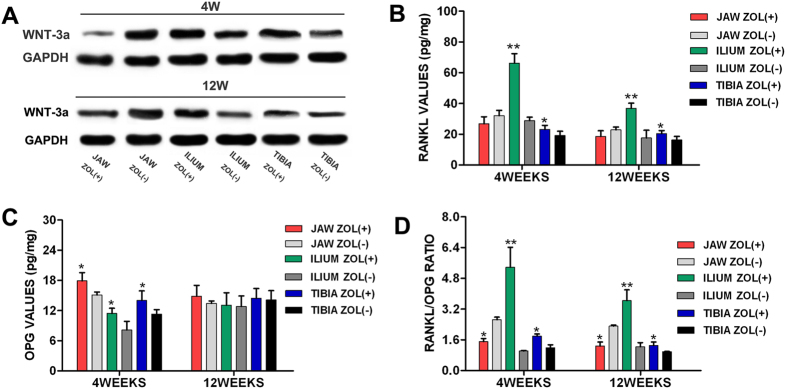
Disparate effects of Zol on osteogenic-related cytokine expressions of jaw, ilium and tibia following injury in a rat model. (**A**) Expression of Wnt-3a in jaw, ilium and tibia of Zol-treated rats at 4 and 12 weeks following injury, as shown by western blot assay. Rats with no Zol treatment served as controls. (**B**–**D**) RANKL expression (**B**), OPG expression (**C**) and RANKL/OPG ratio (**D**) in jaw, ilium and tibia of Zol-treated rats at 4 and 12 weeks following injury, as shown by ELISA assay. *p < 0.05, **p < 0.01.

Western blot results (Fig. 3A) exhibited a significantly decreased level of Wnt-3a in the experimental jaw bone at 4 and 12 weeks after surgery when compared with the control group (p < 0.05), while exhibited a significantly increased level in the experimental iliac and tibial bones (p < 0.05). The expression of RANKL (Fig. 3B) was slightly decreased in jaw bone of Zol-treated rats at 4 and 12 weeks after surgery compared with the untreated rats, but was markedly increased in iliac and tibial bones of Zol-treated rats (p < 0.05). Zol-treated rats showed an increased OPG level (Fig. 3C) in the jaw and peripheral bones at 4 weeks after surgery compared with the respective untreated rats (p < 0.05), while showed no statistically difference at 12 weeks after surgery. Consequently, the ratio of RANKL/OPG (Fig. 3D) was markedly decreased in the experimental jaw bone at 4 and 12 weeks after surgery when compared with the control group (p < 0.05), while significantly increased in the experimental iliac and tibial bones (p < 0.05). These findings suggested that Zol could downregulate RANKL/OPG ratio and Wnt-3a expression in the jaw bone of Zol-treated rats following injury, but upregulate in the peripheral bones, indicating that the deficiency of RANKL/OPG ratio and Wnt-3a expression in the jaw bone of Zol-treated rats may potentially associate with the development of BRONJ.

### Morphological characteristics and identification of BMSCs

**Figure 4:**
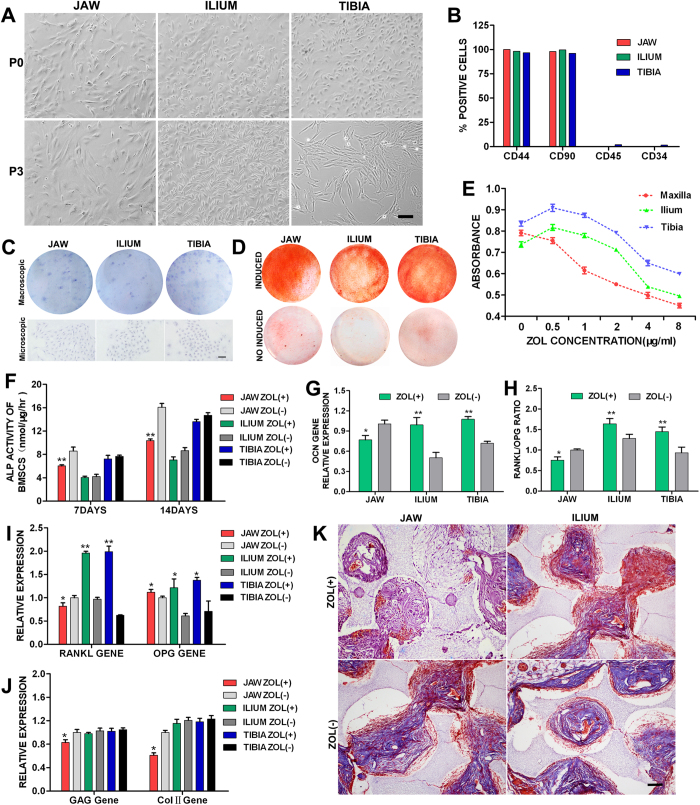
Disparate effects of Zol on osteogenic property of BMSCs derived from jaw, ilium and tibia. (**A**) The morphological characteristics. Scale bar = 100 μm. (**B**) Flow cytometric analysis. (**C**) Representative macroscopic and microscopic images of BMSC colonies after 14 days of culture in petri dishes. Scale bar = 100 μm. (**D**) Representative macroscopic images of Alizarin Red staining after 21 days of osteogenic induction. The cells of each BMSC type without induction served as controls. (**E**) Dose response of each BMSC type to Zol after 4 days exposure. (**F**–**H**) ALP activity (**F**), OCN expression (**G**), RANKL and OPG expression (**I**), RANKL/OPG ratio (**H**), GAG and Col II expression (**J**) of each BMSC type after osteogenic or chondrogenic induction. *p < 0.05, **p < 0.01. (**K**) Representative histological images of MT staining of cell-scaffold complexes at 6 weeks after implantation in a nude mouse model. Scale bar = 100 μm.

The morphology of BMSCs derived from three distinct skeletal sites was observed under an inverted phase contrast microscope. The morphology and size of primary cultured BMSCs were altered gradually with passage. As shown in Fig. 4A, the primary BMSCs derived from iliac and tibial bones exhibited similar morphology. The cells were generally triangular or short spindle shape, and very small in size. However, the primary jaw BMSCs showed a long spindle-like shape, and appeared larger and extended better than the primary peripheral bone BMSCs. Cells proliferated rapidly and became larger as the passages increased, and the morphology tended to be a unanimous appearance. The 3^rd^ generation BMSCs derived from iliac and tibial bones arranged regularly and primarily appeared as slender spindle-like shape. While the 3^rd^ generation jaw BMSCs extended from long spindle-like to polygon shape, and appeared larger and extended better. With increased number of passages, the cell proliferation ability declined. Therefore, the 3^rd^ generation BMSCs with high purity and stable property were utilized for subsequent study.

To identify the isolated BMSCs, flow cytometric analysis, colony forming unit assay and Alizarin Red staining were determined. For each BMSC type, as shown by flow cytometric analysis (Fig. 4B), more than 95% of the same cell population expressed the recognized BMSC markers CD44 and CD90, and lacked expression (<5%) of hematopoietic markers, including CD45 and CD34, suggesting that the obtained BMSCs had the characteristic of high purity. The proliferative capacity of BMSCs was evaluated by colony forming unit assay. Macroscopically, BMSC colonies (see Supplementary Information) were observed in all three stem cell populations after 14 days of culture in petri dishes (Fig. 4C). The colony forming efficiency of each cell population was calculated and depicted as the percentage of the number of cells initially seeded that give rise to visible colonies. The colony forming efficiency of BMSCs derived from jaw, ilium and tibia, was 14.5%, 11.5% and 18.0%, respectively. These data demonstrate the self-renewing capacity of the isolated BMSCs derived from three distinct skeletal sites. Osteogenic differentiation was defined by the appearance of Alizarin Red-positive stained calcium nodules (Fig. 4D). Positive staining for each BMSC type was observed after 21 days of osteogenic induction, while no obvious Alizarin Red-positive matrix was found in uninduced cells. The percentage of the mineralized area over total area of BMSCs derived from jaw, ilium and tibia, was 67.1%, 41.3% and 73.5%, respectively. These findings suggest that the isolated BMSCs had the characteristic to maintain the potential to differentiate into osteoblasts for each BMSC type.

### Disparate proliferation, differentiation potential and bone formation capacity of Zol-treated BMSCs from jaw and peripheral bones

Dose response curves revealed that Zol inhibited proliferation of jaw BMSCs in a dose-dependent way with a concentration in the range from 0.5 to 8 μg/ml (Fig. 4E). However, the proliferation of iliac and tibial BMSCs increased initially at low concentration of Zol (0.5 and 1.0 μg/ml) before a dose-dependent decrease (Fig. 4E). These results suggest that jaw BMSCs are more susceptible to Zol than iliac and tibial BMSCs. 1.0 μg/ml Zol exerted a significant inhibitory effect on jaw BMSCs proliferation, while revealed a slight promotion effect on iliac and tibial BMSCs proliferation. These findings are consistent with the above *in vivo* datasets. Therefore, Zol concentration of 1.0 μg/ml was selected for subsequent study. ALP assay showed that 1.0 μg/ml Zol significantly reduced ALP activity in jaw BMSCs after 7 and 14 days of osteogenic induction (p < 0.01), while had no significant effect on ALP activity in both iliac and tibial BMSCs (Fig. 4F). RT-PCR analysis showed that 1.0 μg/ml Zol inhibited mRNA expression of oteocalcin (OCN) and RANKL, and down-regulated ratio of RANKL/OPG in jaw BMSCs after 14 days of osteogenic induction (P < 0.05) (Fig. 4G,H,I). On the contrary, this concentration of Zol promoted mRNA expression of OCN and RANKL, and up-regulated ratio of RANKL/OPG in iliac and tibial BMSCs (P < 0.05) (Fig. 4G,H,I). OPG mRNA levels were significantly higher in jaw, iliac and tibial BMSCs after exposure to Zol than in their respective control cells (P < 0.05) (Fig. 4I). Moreover, 1.0 μg/ml Zol inhibited chondrogenic related gene expression of glycosaminoglycan (GAG) and type II collagen (Col-II) in jaw BMSCs after 14 days of chondrogenic induction (P  <  0.05), but not in iliac and tibial BMSCs (Fig. 4J). These data suggested that 1.0 μg/ml Zol inhibited osteogenic and chondrogenic differentiation of jaw BMSCs, but had promotional or no significant effect on the differentiation potential of iliac and tibial BMSCs.

Histological analysis showed that cell-scaffold complexes were obviously degraded and absorbed, and numerous collagen fibers (blue-stained) and new bone-like tissues (red-stained) were visible in complexes of untreated control jaw and iliac BMSCs, manifested as no significant difference between the two control groups (Fig. 4K). Zol-treated jaw BMSCs complex presented less scaffold degradation, less collagen fibers and new bone-like tissue formation compared with the untreated group, while Zol-treated iliac BMSCs complex exhibited more new bone-like tissue formation (red-stained) than the respective control group (Fig. 4K). These evidences suggested that Zol inhibited *in vivo* bone formation of jaw BMSCs, but slightly enhanced ectopic bone formation of iliac BMSCs.

Taken together, Zol treatment of jaw and peripheral bone BMSCs showed differences in survival, ALP activity, expression of osteogenic and chondrogenic related genes, and *in vivo* bone formation, suggesting the possibility of dysregulation of bone homeostasis in the jaw bone after exposure to Zol that subsequently may lead to the development of BRONJ.

## Discussion

BRONJ has been described as a serious skeletal complication correlated with long-term oral or intravenous use of nitrogen-containing BPs in patients being treated for malignant disease. Despite the clinical correlation between BPs treatment and BRONJ development, a definitive causal relationship has not yet been established. Therefore, animal models which mimic the hallmarks of the human BRONJ are urgently needed to decipher the underlying pathogenesis and to identify novel therapies for this disease. Several important risk factors for the development of BRONJ have been identified, including the type, administration route and total cumulative dose of BP, history of invasive dental procedures, and development of oral infection. Nitrogen-containing BPs are considered to be more efficacy and toxicity than non-nitrogen-containing BPs, and are at higher risk for developing BRONJ (primarily Zol and PA)^28^,^29^,^30^. The BRONJ risk with Zol was 5–10 fold higher than with PA alone^28^,^30^, and the BRONJ lesions also developed earlier in patients receiving Zol^29^. Moreover, intravenous BPs for malignant disease are usually more likely to develop BRONJ than oral BPs for osteoporosis based on epidemiologic data. The cumulative incidence of BRONJ in oncologic patients receiving intravenous BPs ranges from 0.8% to 12%^6^, whereas in osteoporotic patients taking oral BPs, it ranges from 0.01% to 0.04%^31^. It is also recognized that 60–70% of BRONJ cases occurred in patients following routine dental surgical procedures (primarily tooth extraction)^7^,^8^, and dental surgery increased the risk of BRONJ about 10–40 fold^32^. Invasive dental procedures were thus considered to be the most common predisposing factor for developing BRONJ, and patients accompanied with oral inflammatory diseases^33^,^34^ (eg. periodontitis, dental abscess) are at increased risk for developing BRONJ. Therefore, we induced a rat BRONJ model by intravenous administration of Zol undergoing tooth extraction. Recognizing that oral bacterial infection has an effect on the progression of BRONJ, the socket gingiva was tightly sutured for preventing infection in the generation of our rat BRONJ-like model.

In this study, rat BRONJ-like lesions showed similar characteristics to human disease involving open sockets with unhealed mucosa, necrotic bone or sequestra, focal osteosclerosis, and strong inflammatory infiltration, as demonstrated by clinical, histological and radiographic examination. In Zol-treated rats, all developed osteonecrosis at the extraction sites and 44% (4/9) rats failed to remodel up to 12 weeks, exhibiting open sockets with exposed necrotic bone and no mucosal coverage. The clinical manifestations of BRONJ-like lesions, specifically the presence of soft tissue defects, persisted for more than eight weeks and therefore met the current clinical diagnostic criteria for human BRONJ. Although the alveolar bone was covered by intact mucosa at 12 weeks after extraction in 56% (5/9) of Zol-treated rats, the epithelium was completely separated from the underneath necrotic bone which was infiltrated by numerous inflammatory cells and bacterial colonies. Therefore, Zol induced impaired bone healing rather than just a delay in bone remodeling. The persistent presence of typical histological and radiographic hallmarks of BRONJ supports our model of BRONJ-like disease in rats, and allows for defining the pathogenesis of the disease at the cellular and molecular levels, leading to better diagnosis and more targeted treatments. Retrospective analysis of human BRONJ cases shows BRON occurs primarily in jaw bones not in peripheral bones, yet the exact mechanism by which this occurs remains unknown. To verify that BRONJ is a site-specific osseous pathology, we determine the potential systemic effect of Zol on bone remodeling of iliac and tibial bones following injury in the same individuals. We demonstrate that Zol treatment suppresses jaw bone remodeling after tooth extraction, and induces BRONJ-like lesions in rats that faithfully recapitulates typical histological and radiographic hallmarks of the human BRONJ, whereas enhances peripheral bone quantity in bone remodeling following injury in the same individuals, shown as increased cortical bone thickness, increased trabecular bone formation and accelerated bone repair. Consistent with clinical epidemiological data, we showed that the necrosis occurred only in the jaw bones, suggesting that BPs might modulate bone remodeling in a skeletal site-specific pattern.

In our model, we observed BRONJ-like lesions occurred exclusively at the extracted sites, and the distal sites or the contralateral sites without bone defect were not affected. In contrast, no necrotic lesions were observed at the defect sites of the peripheral bones. These findings suggest that bone trauma is an important predisposing factor rather than a crucial factor for developing BRONJ. Moreover, we observed that necrotic bone was predominantly found adjacent to area of intense local inflammatory infiltration (Fig. 1D–F), suggesting that inflammation reaction may potentially contribute to an increased risk for necrosis in BRONJ-like disease. However, it is still unknown whether the infection plays a causative role in the pathogenesis of BRONJ or merely represents secondary injury. Further studies are needed to verify whether jaw necrosis arises prior to the infection in the bone or soft tissue.

Because of the uncertainty regarding the exact pathogenic mechanism of BRONJ, the effects of nitrogen-containing BPs on bone remodeling should be evaluated at the cellular and particularly molecular level. We explore the changes in cytokine expression of jaw and peripheral bones after Zol treatment. RANKL/RANK/OPG system is capable of regulating the dynamic balance between osteoclastic bone resorption and osteoblastic bone formation and thereby maintaining bone homeostasis and bone mass^35^. RANKL and OPG, expressed by osteoblasts or BMSCs, have an antagonistic effect on osteoclastogenesis and osteoclast activation^35^. RANKL, a type II membrane protein that belongs to the tumor necrosis factor (TNF) superfamily, is essential for osteoclast differentiation, activation, and survival, and consequently promotes bone resorption^36^. OPG acts as a decoy receptor by sequestering RANKL therefore inhibiting osteoclast-mediated bone resorption^36^. The RANKL/OPG ratio is thus an important determinant of bone mass in normal bone remodeling. The canonical Wnt/β-catenin signaling pathway, an important regulatory pathway for bone metabolism, plays a crucial role in modulating bone formation and bone resorption and thereby affecting bone remodeling^37^,^38^. Wnt-3a regulates the nuclear accumulation of β-catenin and Lef/Tcf-sensitive transcription of developmentally important genes via the canonical Wnt/β-catenin pathway. Evidences have showed that overexpression of Wnt-3a promoted osteoblast precursors proliferation and enhanced ALP activity^39^,^40^,^41^, the early osteogenic differentiation marker, by activating the canonical Wnt/β-catenin pathway, suggesting its osteogenic potency to form new bone *in vivo*. Wnt-3a is capable of promoting the osteoblastic differentiation of BMSCs, and thus identified as a regulator of bone mass and bone remodeling. In our study, we showed that Zol downregulated RANKL/OPG ratio and Wnt-3a expression in the jaw bones of Zol-treated rats at 4 and 12 weeks after surgery, but induced opposite effects in the peripheral bones. Bone tissue is continuously remodelled, and bone mass is maintained by the dynamic balance between bone resorption and bone formation. Zol inhibits osteoclast-mediated bone resorption in the jaw bones after tooth extraction by down-regulating RANKL/OPG ratio and RANKL/RANK/OPG signaling pathway, and also suppresses osteoblast-mediated bone formation by down-regulating Wnt-3a and the canonical Wnt/β-catenin pathway. We speculate that this marked decrease in both bone resorption and bone formation results in the oversuppression of bone turnover, and ultimately may lead to the failure of bone remodeling and the development of BRONJ after tooth extraction. On the other hand, the up-regulation of Wnt-3a in the peripheral bones promotes BMSCs osteoblastic differentiation and bone formation by activating the canonical Wnt/β-catenin pathway, meanwhile, the up-regulation of RANKL/OPG ratio enhances osteoclast differentiation and bone resorption by activating RANKL/RANK/OPG signaling pathway. This active bone remodeling may result in consecutive positive bone turnover, and consequently promote physiological bone remodeling in the peripheral bones after surgery. Therefore, the deficiency of RANKL/OPG ratio and Wnt-3a expression at the extracted alveolar sites after exposure to Zol might be implicated as predisposing factors for development BRONJ.

Developmentally, jaw bones originate exclusively from neuroectoderm and undergo primarily intramembranous ossification, whereas peripheral bones develop from mesoderm and are formed by both endochondral and intramembranous ossification^24^,^25^,^26^. In the process of bone remodeling after injury, osteoclasts, the cells responsible for bone resorption, are derived from the mononuclear phagocytic system, while osteoblasts for bone formation are derived from the BMSCs in their respective tissues. Thus, functional difference in bone remodeling following injury may exist at different anatomic sites. Studies have proved that cell differentiation and function of jaw bone BMSCs and peripheral bone BMSCs is different^27^. To test the hypothesis that osteogenic response of BMSCs to BPs is skeletal site-dependent, we compared the *in vitro* proliferation and differentiation capacity of jaw bone vs. peripheral bone BMSCs after exposure to BPs, as well as the *in vivo* bone formation capacity. Our results showed that Zol affected proliferative capacity and differentiation potential of jaw bone BMSCs differently from those of peripheral bone BMSCs. The overall survival of jaw bone BMSCs decreased with the increase of Zol concentration, whereas the cell proliferation of iliac and tibial BMSCs increased initially before a dose-dependent decrease, indicating that jaw bone BMSCs are more susceptible to Zol than peripheral bone BMSCs. Jaw bone BMSCs showed a weakened osteogenic and chondrogenic potential after exposure to Zol based on ALP activity and gene expression of OCN, GAG and Col**II**, and also exhibited a decreased capacity to induce bone regeneration *in vivo*. On the contrary, Zol had no inhibitory or a slight promotion effect on differentiation potential and *in vivo* bone forming capacity of peripheral bone BMSCs. We also demonstrated that Zol down-regulated the ratio of RANKL/OPG in jaw bone BMSCs, but up-regulated in peripheral bone BMSCs, which is consistent with the *in vivo* data described above. The distinct dissimilarities in proliferation, ALP activity, the expression of osteogenic and chondrogenic related marker genes and the *in vivo* bone formation capacity of jaw and peripheral bone BMSCs after exposure to Zol may help explain the differences in the vivo bone remodeling observed in jaw bones when compared with peripheral bones.

In conclusion, we find that Zol treatment suppresses alveolar bone remodeling after tooth extraction and leads to BRONJ-like disease in rats, whereas enhances peripheral bone quantity in bone remodeling following injury in the same individuals. The distinct dissimilarities in osteogenic and chondrogenic response of jaw and peripheral bone BMSCs to Zol could be implicated as a potential etiological factor for development BRONJ. This rat model of BRONJ-like disease will allow for evaluating different risk factors in the development of BRONJ and deciphering the underlying pathogenesis of the disease, leading to effective prevention, timely diagnosis and precisely targeted treatments in human patients. Moreover, the deficiency of RANKL/OPG ratio and Wnt-3a expression at the extracted alveolar sites after exposure to Zol may help explain the pathophysiology of BRONJ, suggesting that the design of novel drugs that targets the RANKL/RANK/OPG signaling pathway and the canonical Wnt/β-catenin pathway could potentially revolutionize treatment for BRONJ.

## Methods

### Animals

Female Sprague-Dawley rats (8–10 weeks old, weighing 200–250 g), purchased from Shanghai SLAC Laboratory Animal Co. Ltd (Shanghai, China), were used in this experiment. Rats were kept in a standard animal room at a temperature of 22 ± 1 °C, a relative humidity of 55 ± 10%, and a 12-hour light-dark cycle, where they had free access to food and water *ad libitum.* This study was performed in accordance with the guidelines and regulations for the care and use of laboratory animals of the National Institutes of Health. All procedures were approved by the Institutional Animal Care and Use Committee of Tongji University (Shanghai, China).

### Surgery and experimental procedures

**Figure 5:**
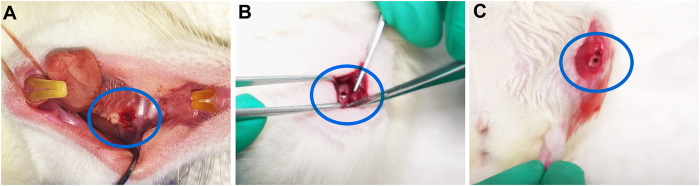
Creation of bone defects on jaw, ilium and tibia in Sprague-Dawley rats. (**A**) Maxillary or mandibular first molar was extracted at 2 weeks after administration of intravenous Zol. (**B**,**C**) Drill hole defects were created on the ipsilateral ilium (**B**) and tibia (**C**).

Rats were treated intravenously with Zol (80 μg/kg body weight per week) via the tail vein. Two weeks after administration, first molar from either maxilla or mandible was extracted (Fig. 5A) using a dental luxator under general anaesthesia by intraperitoneal injection of ketamine hydrochloride (100 mg/kg) and xylazine (5 mg/kg). Subsequently, drill hole defect (Diameter: 2 mm; Depth: 1mm) was prepared on the ipsilateral ilium and tibia under continuous irrigation with saline solution (Fig. 5B,C). Haemostasis was achieved using sterile gauze and the surgical site was sutured with resorbable sutures. After surgical operations, rats were given intramuscular injection of penicillin (500,000 U/kg) for three days to prevent infection. Drugs were continuously administered for 1 to 12 weeks. Thereafter, rats were sacrificed by parcel, and the intact maxillas or mandibles, tibias, ilia were harvested. Untreated rats with the same surgical procedures were used as controls.

### Histological observation

The harvested specimens were fixed in 4% paraformaldehyde at 4 °C for 48 h and then decalcified in a 10% EDTA solution at room temperature (RT) for 4 to 6 weeks. After dehydration with gradient ethanol, the specimens were degreased in xylene and then embedded in paraffin. Sections (5 μm thick) were analyzed histochemically by haematoxylin-eosin (HE) and Masson’s trichrome (MT) staining according to the manufacturer’s instructions. Osteoclasts were identified by tartrate resistant acid phosphatase (TRAP) staining using an acid phosphatase leukocyte kit (Sigma-Aldrich, USA), following the protocols recommended by the manufacturer. The extent of osteonecrosis was quantified as described in previous studies^42^. Five uniformly spaced HE-stained slides of the extraction site from each specimen were scanned digitally by a ScanScope slide scanner. An area of interest (~4.0 mm^2^) located within 2.0 mm of the second molar was selected. The necrotic bone, defined as any region that contains three or more empty lacunae per 1000 μm^2^, was marked. The total areas of necrotic bone were analyzed for each slide using ImageScope software. An average value of necrotic bone area was calculated over the above fives slides per rat, and then the percentage of necrotic bone area over the total bone area was calculated.

### Microcomputed Tomography Analysis

Rat specimens were scanned and reconstructed with 10 μm isotropic voxels on a μCT system (Scanco Medical, Zurich, Switzerland). The three-dimensional images were reconstructed using analysis sofeware(Start Xming). Three regions of surgical sites were selected for bone morphometric analysis. Bone volume fraction (BV/TV) was calculated as a percentage by dividing the area occupied by the mineralized bone over the total volume (TV).

### Cytokine analysis

Expression of RANKL, OPG and Wnt-3a in bone specimens from three distinct skeletal sites was investigated. The extraction site of maxilla or mandible, the surgical site of ilium and tibia, were dissected and frozen in liquid nitrogen immediately. RANKL and OPG concentrations were determined using Enzyme-linked immunosorbent assay (ELISA). Specimens were ground into pieces in ice-cold sterile PBS supplemented with complete protease inhibitor cocktail, followed by homogenization using a rotor-stator homogenizer. The homogenates were centrifuged (12000 g, 5 min, 4 °C) and the supernatants were then transferred to fresh precooled eppendorf tubes. The amounts of RANKL and OPG were determined using commercially available rat ELISA kits (Uscn Life Inc., Wuhan, China) according to the manufacturer’s instructions.

Wnt-3a expression was measured by western blot analysis. Anti-Wnt3a monoclonal antibody was used as primary antibody, and GAPDH served as an internal control. The shattered bones lysed in RIPA buffer supplemented protease inhibitors at 4 °C for 1 h. The samples were then homogenized, centrifuged, and treated as described above. Total protein concentrations were measured using the BCA Protein Assay Kit (Beyotime, Shanghai, China). Equivalent amounts of protein derived from the collected supernatants were subjected to 12% SDS-PAGE, followed by transferring onto nitrocellulose membranes. The membranes were blocked with the blocking buffer (Tris-buffered saline, containing 0.1% Tween-20 and 5% non-fat milk) for 1 h at RT, and incubated with primary antibody at 4 °C overnight. After extensive washing, the membranes were incubated with secondary antibody conjugated to horseradish peroxidase (HRP). The proteins were visualized using the Immune-Star Western C Chemiluminescence Kit (Bio-Rad, California, USA). The intensity of the bands was quantified using ImageJ software, and normalized by GAPDH expression levels.

### BMSCs isolation and culture

For isolation of jaw BMSCs, the neonatal female SD rats were euthanized, and then mandibles and maxillas were dissected. Subsequently, soft tissues were scraped off completely, and the third molars were removed. After puncture with a 21-gauge spinal needle on the buccal cortex, the mandibular and maxillary bone marrow was flushed out with Dulbecco’s modified Eagle medium (DMEM), and cell suspensions were collected from the extraction socket^43^. Peripheral bone BMSCs were simultaneously isolated from bone marrow of ilia and tibias as reported previously^44^. Harvested cells from three distinct skeletal sites were respectively pooled, filtered through a 40-μm-pore-size cell strainer filter, and incubated at 37 °C in a humidified atmosphere of 5% CO_2_. Primary culture of rat BMSCs were cultured in DMEM supplemented with 10% fetal bovine serum (FBS), 100 IU/ml penicillin and 100 mg/ml streptomycin, and the culture medium was changed every 3 days.

### Identification of BMSCs

The specific surface antigens of the 3^rd^ generation BMSCs isolated from three distinct skeletal sites were identified by flow cytometry analysis. Briefly, BMSCs were lightly trypsinized, washed with ice-cold PBS supplemented with 0.5% bovine serum albumin (BSA), and then centrifuged for 5 min at 250 g. Afterward, cells were resuspended and incubated with fluorescein isothiocynate (FITC) conjugated antibodies against rat CD34, CD44, CD45, and CD90 for 30 min at 4 °C. BMSCs incubated without any target were used as a negative control. Data were acquired and analyzed using a flow cytometer equipped with FlowJo software.

The proliferation potential and colony forming efficiency of stem cells derived from different origins was determined by colony forming unit (CFU) assay. In brief, BMSCs from primary cultures were trypsinized and inoculated at a density of 200 cells/well in 6-well plates. 14 days after incubation, colonies were fixed in ice-cold 4% paraformaldehyde for 15 min and then stained with Giemsa solution for 15 min at RT. The BMSC-derived CFUs, consisting of at least 50 cells, were enumerated by microscopy, and the total colony forming efficiency (CFE) of the same cell population was calculated by dividing the number of colonies by the number of inoculated cells.

Extracellular matrix mineralization of stem cells was also identified with Alizarin red staining of calcified nodules. The 3^rd^ generation BMSCs were incubated at a seeding density of 10^4^ cells/cm^2^ in 6-well plates. Upon reaching confluency, the growth medium was replaced with osteogenic induction medium (basal culture medium supplemented with 4 mM β-glycerophosphate, 100 nM dexamethasone, 0.17 mM L-ascorbic acid-2-phosphate, and 2 mM L-glutamine). Cells were cultured in osteogenic induction medium for 21 days, then cells were fixed and stained with 0.2% alizarin red S (pH 8.3) for 1 h at RT. Finally, cells were washed with deionized water, dried and photographed by microscopy (Nikon, Japan). Alizarin red S staining was quantified as the percentage of the mineralized area over the total area using ImageScope software.

### Dose response and cell proliferation

Dose response of each BMSC type to Zol was determined using the AlamarBlue Cell Viability Reagent (Invitrogen, California, USA). Trypsinized cells were seeded at a density of 10^4^ cells/cm^2^ in 96-well plates and cultured in basal culture medium for 24 h. Cells were subsequently incubated in solutions of various concentrations of Zol (0, 0.5, 1.0, 2.0, 4.0 and 8.0 μg/ml). After 4 days exposure to Zol, metabolically active cells were determined by a cell viability assay following the manufacturer’s instructions. Briefly, the medium was discarded and replaced with fresh culture medium supplemented with 10% cell viability reagent. Afterward, cells were incubated at 37 °C for 4 h. The optical density was measured using a spectrophotometer. Subsequently, 1.0 μg/ml of Zol was performed in experiments described below.

### Osteogenic and chondrogenic differentiation potential

To evaluate osteogenic potential of Zol-treated BMSCs from jaw and peripheral bones, ALP activity, a significant indicator of early osteogenesis, was measured by quantitative ALP activity assay. The 3^rd^ generation BMSCs were seeded at a density of 10^4^ cells/cm^2^ in 24-well plates. After 24 h of culture, the medium was replaced with osteogenic induction medium containing 1.0 μg/ml of Zol, and the cells were subsequently cultured for 7 or 14 days. Cells cultured in standard osteogenic induction medium were used as controls. After removing culture medium and washing with PBS, cells were lysed using CelLytic™ P Cell Lysis Reagent (Sigma-Aldrich, USA) for 15 min at 4 °C. The cell lysate was centrifuged (12000g, 5 min, 4 °C) and the supernatants were then transferred to fresh precooled eppendorf tubes. ALP activity was measured using an ALP activity assay kit (Wako, Japan) according to the manufacturer’s protocol. Total protein was quantified by a BCA Protein Assay Kit (Beyotime, Shanghai, China), and ALP activity was expressed as nanomoles of p-nitrophenol liberated per microgram of total cellular protein per hour.

OCN is a non-collagenous extracellular matrix protein in bone and a late marker of osteoblast differentiation. Col-II and GAG are major and essential components of the cartilage extracellular matrix and specific markers of cartilage differentiation. The mRNA expression of osteogenic and chondrogenic related genes was analyzed using quantitative real time polymerase chain reactions (qRT-PCR) technique (see Table 2). The 3^rd^ generation BMSCs derived from three distinct skeletal sites were seeded in 24-well plates as above. After 24 h of culture, the medium was replaced with osteogenic (basal DMEM medium, 4 mM β-glycerophosphate, 100 nM dexamethasone, 0.17 mM L-ascorbic acid-2-phosphate, and 2 mM L-glutamine) or chondrogenic (high-glucose DMEM, 10% FBS, 100 IU/ml penicillin, 100 mg/ml streptomycin, ITS × 1, 1 mM sodium pyruvate, 0.35 mM L-prolin, 0.17 mM L-ascorbic acid-2-phosphate, 100 nM dexamethasone, and 10 ng/mL TGF-β3) induction medium containing 1.0 μg/ml of Zol, and the cells were subsequently cultured for 14 days. Cells without Zol exposure were used as controls. Total cellular RNA was extracted from cell lysates using Trizol reagent following the manufacture’s recommendation. Equivalent quantities of eluted RNA were reverse transcribed using a First-Strand cDNA Synthesis Kit (Toyobo, Japan). Then, the qRT-PCR was performed in triplicate on a real time PCR system (Bio-Rad, California, USA) using SYBR Green Master Mix (Thermo, USA). The gene expression level relative to endogenous control GAPDH was calculated using the 2−ΔΔCT formula.

### Bone Regeneration

Bone formation capacity of Zol-treated BMSCs from jaw and iliac bones was evaluated in a nude mouse model via ectopic implantation following Tongji University’s approved animal care protocol. BMSC/Bio-Oss complex, consisting of 1 × 10^5^ Zol-treated or untreated BMSCs attached to 40 mg Bio-Oss (particle size 0.25–1.0 mm, Geistlich, Switzerland) was transplanted into subcutaneous tissue of nude mice. After 6 weeks, transplants were harvested, fixed, decalcified, paraffin-embedded, sectioned, and MT-stained. The photomicrographs were taken using a Leitz microscope (Leitz, Germany) equipped with a Nikon digital camera (Nikon, Japan).

### Statistical Analysis

Data were expressed as mean ± standard deviation (mean ± sd). All experiments were performed independently in triplicate. Student’s t-test was employed for statistical analysis. A value for p < 0.05 was considered to be statistically significant, and a value for p < 0.01 was considered to be highly statistically significant.

## Additional Information

**How to cite this article**: Gong, X. *et al*. Skeletal Site-specific Effects of Zoledronate on *in vivo* Bone Remodeling and *in vitro* BMSCs Osteogenic Activity. *Sci. Rep.* **7**, 36129; doi: 10.1038/srep36129 (2017).

**Publisher's note:** Springer Nature remains neutral with regard to jurisdictional claims in published maps and institutional affiliations.

## Supplementary Material

Supplementary Data

## Figures and Tables

**Table 1:** Bone remodeling of jaw and peripheral bones in Zol-treated rats.

	Necrotic bone affected (total)	Inflammation affected (total)	Soft tissue defects affected (total)
Jaw Zol (+)	9 (9)	7 (9)	4 (9)
Jaw Zol (−)	0 (9)	0 (9)	0 (9)
Ilium Zol (+)	1 (9)	1 (9)	0 (9)
Ilium Zol (−)	0 (9)	0 (9)	0 (9)
Tibia Zol (+)	0 (9)	0 (9)	0 (9)
Tibia Zol (−)	0 (9)	0 (9)	0 (9)

**Table 2:** Primer sequences for RT–PCR analysis.

Gene	Sense primer (5′~3′)	Antisense primer (3′~5′)
OCN	TCTCTGACCTCACAGATGCCAA	ACCGTAGATGCGTTTGTAGGC
RANKL	ATGGAAGGCTCATGGTTGGAT	ACAGGTAATAGAAGCCATCTTGGTT
OPG	GTGAAGACACACTACCTGACTCCTG	GCAGCATTGATGGTGAGGTG
Col **II**	GGGAATGTCCTCTGCGATGAC	CAACCCCGAGATCCCCTTC
GAG	CCTGCTACTTCATCGACCCC	AGATGCTGTTGACTCGAACCT
GAPDH	TATGTCGTGGAGTCTACTGGT	GAGTTGTCATATTTCTCGTGG

## References

[b1] Eastell R., Walsh J. S., Watts N. B. & Siris E. Bisphosphonates for postmenopausal osteoporosis. Bone. 49, 82–88 (2011). 21349354 10.1016/j.bone.2011.02.011

[b2] Reid I. R. . A single infusion of zoledronic acid produces sustained remissions in Paget disease: data to 6.5 years. J Bone Miner Res. 26, 2261–2270 (2011). 21638319 10.1002/jbmr.438

[b3] Drake M. T., Clarke B. L. & Khosla S. Bisphosphonates: mechanism of action and role in clinical practice. Mayo Clin Proc. 89, 1032–1045 (2008). 10.4065/83.9.1032 PMC2667901 18775204

[b4] Marx R. E. Pamidronate (Aredia) and zoledronate (Zometa) induced avascular necrosis of the jaws: a growing epidemic. J Oral Maxillofac Surg. 61, 1115–1117 (2003). 12966493 10.1016/s0278-2391(03)00720-1

[b5] Silverman S. L. & Landesberg R. Osteonecrosis of the jaw and the role of bisphosphonates: a critical review. Am J Med. 122, S33–S45 (2009). 10.1016/j.amjmed.2008.12.005 19187811

[b6] Ruggiero S. L. . American Association of Oral and Maxillofacial Surgeons position paper on bisphosphonate-related osteonecrosis of the jaws–2009 update. J Oral Maxillofac Surg. 67, 2–12 (2009). 10.1016/j.joms.2009.01.009 19371809

[b7] Tardast A., Sjöman R., Løes. S. & Abtahi J. Bisphosphonate associated osteomyelitis of the jaw in patients with bony exposure: prevention, a new way of thinking. J Appl Oral Sci. 23, 310–314 (2015). 26221926 10.1590/1678-775720140506 PMC4510666

[b8] Van den Wyngaert T., Huizing M. T. & Vermorken J. B. Bisphosphonates and osteonecrosis of the jaw: cause and effect or a post hoc fallacy? Ann Oncol. 17, 1197–1204 (2006). 16873439 10.1093/annonc/mdl294

[b9] Bodem J. P. . Surgical management of bisphosphonate-related osteonecrosis of the jaw stages II and III. Oral Surg Oral Med Oral Pathol Oral Radiol. 121, 367–372 (2016). 26795450 10.1016/j.oooo.2015.10.033

[b10] Van den Wyngaert T., Claeys T., Huizing M. T., Vermorken J. B. & Fossion E. Initial experience with conservative treatment in cancer patients with osteonecrosis of the jaw (ONJ) and predictors of outcome. Ann Oncol. 20, 331–336 (2009). 18953067 10.1093/annonc/mdn630

[b11] Silva M. L. . Effect of hyperbaric oxygen therapy on tooth extraction sites in rats subjected to bisphosphonate therapy-histomorphometric and immunohistochemical analysis. Clin Oral Investig. (2016). 10.1007/s00784-016-1778-3 26955837

[b12] Yuan H. . Revival of nitrogen-containing bisphosphonate-induced inhibition of osteoclastogenesis and osteoclast function by water-soluble microfibrous borate glass. Acta Biomater. 31, 312–325 (2016). 26678828 10.1016/j.actbio.2015.12.009

[b13] Takaoka K. . Establishment of an Animal Model of Bisphosphonate-Related Osteonecrosis of the Jaws in Spontaneously Diabetic Torii Rats. PLoS One 10, e0144355 (2015). 26659123 10.1371/journal.pone.0144355 PMC4684366

[b14] Odvina C. V. . Severely suppressed bone turnover: a potential complication of alendronate therapy. J Clin Endocrinol Metab. 90, 1294–1301 (2005). 15598694 10.1210/jc.2004-0952

[b15] Sharma D., Hamlet S. M., Petcu E. B. & Ivanovski S. The effect of bisphosphonates on the endothelial differentiation of mesenchymal stem cells. Sci Rep. 6, 20580 (2016). 26857282 10.1038/srep20580 PMC4746673

[b16] Ferretti G. . Zoledronic-acid-induced circulating level modifications of angiogenic factors, metalloproteinases and proinflammatory cytokines in metastatic breast cancer patients. Oncology. 69, 35–43 (2005). 16088233 10.1159/000087286

[b17] Soydan S. S. . Effects of alendronate and pamidronate on apoptosis and cell proliferation in cultured primary human gingival fibroblasts. Hum Exp Toxicol. 34, 1073–1082 (2015). 25636638 10.1177/0960327115569808

[b18] Scheper M. A., Badros A., Chaisuparat R., Cullen K. J. & Meiller T. F. Effect of zoledronic acid on oral fibroblasts and epithelial cells: a potential mechanism of bisphosphonate-associated osteonecrosis. Br J Haematol. 144, 667–676 (2009). 19036117 10.1111/j.1365-2141.2008.07504.x PMC2739302

[b19] Landesberg R. . Inhibition of oral mucosal cell wound healing by bisphosphonates. J Oral Maxillofac Surg. 66, 839–847 (2008). 18423269 10.1016/j.joms.2008.01.026 PMC2426967

[b20] Reid I. R., Bolland M. J. & Grey A. B. Is bisphosphonate-associated osteonecrosis of the jaw caused by soft tissue toxicity? Bone. 41, 318–320 (2007). 17572168 10.1016/j.bone.2007.04.196

[b21] Katsarelis H., Shah N. P., Dhariwal D. K. & Pazianas M. Infection and medication-related osteonecrosis of the jaw. J Dent Res. 94, 534–539 (2015). 25710950 10.1177/0022034515572021

[b22] Crincoli V. . Microbiological investigation of medication-related osteonecrosis of the jaw: preliminary results. J Biol Regul Homeost Agents. 29, 977–983 (2015). 26753664

[b23] Lesclous P. . Bisphosphonate-associated osteonecrosis of the jaw: a key role of inflammation? Bone. 45, 843–852 (2009). 19631301 10.1016/j.bone.2009.07.011

[b24] Chai Y. . Fate of the mammalian cranial neural crest during tooth and mandibular morphogenesis. Development. 127, 1671–1679 (2000). 10725243 10.1242/dev.127.8.1671

[b25] Helms J. A. & Schneider R. A. Cranial skeletal biology. Nature. 423, 326–331 (2003). 12748650 10.1038/nature01656

[b26] Charbord P., Tavian M., Humeau L. & Péault B. Early ontogeny of the human marrow from long bones: an immunohistochemical study of hematopoiesis and its microenvironment. Blood 87, 4109–4119 (1996). 8639768

[b27] Akintoye S. O. . Skeletal site-specific characterization of orofacial and iliac crest human bone marrow stromal cells in same individuals. Bone 38, 758–768 (2006). 16403496 10.1016/j.bone.2005.10.027

[b28] Kos M. Incidence and risk predictors for osteonecrosis of the jaw in cancer patients treated with intravenous bisphosphonates. Arch Med Sci. 11, 319–324 (2015). 25995747 10.5114/aoms.2015.50964 PMC4424249

[b29] Nakamura M. . Analysis of the time-to-onset of osteonecrosis of jaw with bisphosphonate treatment using the data from a spontaneous reporting system of adverse drug events. J Pharm Health Care Sci. 1, 34 (2015). 26819745 10.1186/s40780-015-0035-2 PMC4728763

[b30] Zervas K. . Incidence, risk factors and management of osteonecrosis of the jaw in patients with multiple myeloma: a single-centre experience in 303 patients. Br J Haematol. 134, 620–623 (2006). 16889620 10.1111/j.1365-2141.2006.06230.x

[b31] Mavrokokki T., Cheng A., Stein B. & Goss A. Nature and frequency of bisphosphonate-associated osteonecrosis of the jaws in Australia. J Oral Maxillofac Surg. 65, 415–423 (2007). 17307586 10.1016/j.joms.2006.10.061

[b32] Kos M. Incidence and risk predictors for osteonecrosis of the jaw in cancer patients treated with intravenous bisphosphonates. Arch Med Sci. 11, 319–324 (2015). 25995747 10.5114/aoms.2015.50964 PMC4424249

[b33] Li C. L. . Role of periodontal disease in bisphosphonate-related osteonecrosis of the jaws in ovariectomized rats. Clin Oral Implants Res. 27, 1–6 (2016). 25371026 10.1111/clr.12502

[b34] Thumbigere-Math V. . Periodontal disease as a risk factor for bisphosphonate-related osteonecrosis of the jaw. J Periodontol. 85, 226–233 (2014). 23786404 10.1902/jop.2013.130017 PMC3972496

[b35] Khosla S. Minireview: the OPG/RANKL/RANK system. Endocrinology 142, 5050–5055 (2001). 11713196 10.1210/endo.142.12.8536

[b36] Boyce B. F. & Xing L. Biology of RANK, RANKL, and osteoprotegerin. Arthritis Res Ther. 9, S1 (2007). 17634140 10.1186/ar2165 PMC1924516

[b37] Issack P. S., Helfet D. L. & Lane J. M. Role of Wnt signaling in bone remodeling and repair. HSS J. 4, 66–70 (2008). 18751865 10.1007/s11420-007-9072-1 PMC2504275

[b38] Glass D. A. & Karsenty G. Molecular bases of the regulation of bone remodeling by the canonical Wnt signaling pathway. Curr Top Dev Biol. 73, 43–84 (2006). 16782455 10.1016/S0070-2153(05)73002-7

[b39] Eijken M. . Wnt signaling acts and is regulated in a human osteoblast differentiation dependent manner. J Cell Biochem. 104, 568–579 (2008). 18186078 10.1002/jcb.21651

[b40] Kulkarni N. H. . Orally bioavailable GSK-3alpha/beta dual inhibitor increases markers of cellular differentiation *in vitro* and bone mass *in vivo*. J Bone Miner Res. 21, 910–920 (2006). 16753022 10.1359/jbmr.060316

[b41] Gregory C. A. . How Wnt signaling affects bone repair by mesenchymal stem cells from the bone marrow. Ann N Y Acad Sci. 1049, 97–106 (2005). 15965110 10.1196/annals.1334.010

[b42] Bi Y. . Bisphosphonates cause osteonecrosis of the jaw-like disease in mice. Am J Pathol. 177, 280–290 (2010). 20472893 10.2353/ajpath.2010.090592 PMC2893671

[b43] Aghaloo T. L. . Osteogenic potential of mandibular vs. long-bone marrow stromal cells. J Dent Res. 89, 1293–1298 (2010). 20811069 10.1177/0022034510378427 PMC3113466

[b44] Javazon E. H., Colter D. C., Schwarz E. J. & Prockop D. J. Rat marrow stromal cells are more sensitive to plating density and expand more rapidly from single-cell-derived colonies than human marrow stromal cells. Stem Cells 19, 219–225 (2001). 11359947 10.1634/stemcells.19-3-219

