# Supplementary material for: Skeletal Site-specific Effects of Zoledronate on in vivo Bone Remodeling and in vitro BMSCs Osteogenic Activity
Source: Sci Rep. 2017 Jan 31;7:36129. doi: 10.1038/srep36129 (PMC5282532; doi:10.1038/srep36129)

Skeletal Site-specific Effects of Zoledronate on *in vivo* Bone Remodeling and *in vitro* BMSCs Osteogenic Activity

Xue Gong+, Wanlu Yu+, Hang Zhao, Jiansheng Su*, Qing Sheng

Supplementary FACS data

Ilium BMSCs:

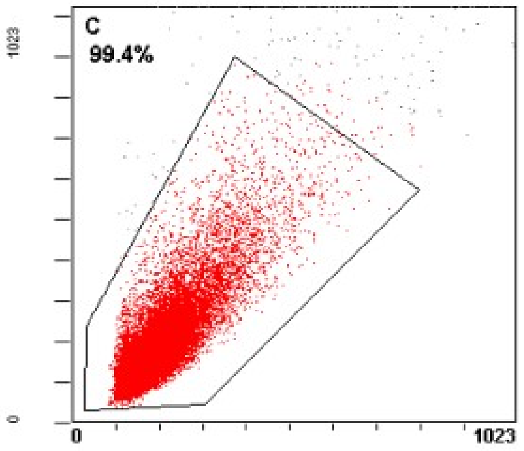

Region	Number	%Total	%Gated	X-Mean	Y-Mean
ALL	34764	100.00	100.00	193	183
C	34570	99.44	99.44	191	179

CD45

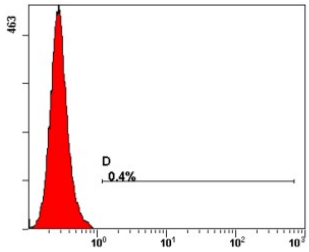

CD34

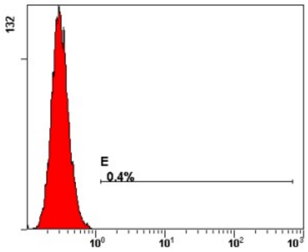

CD44

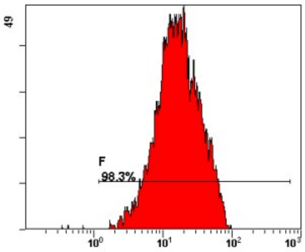

CD90

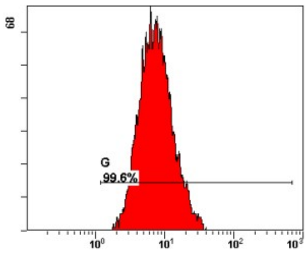

Jaw BMSCs:

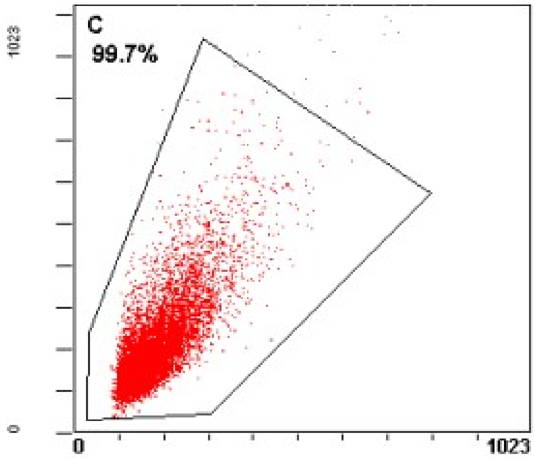

Region	Number	%Total	%Gated	X-Mean	Y-Mean
ALL	10137	100.00	100.00	181	208
C	10102	99.65	99.65	180	206

CD45	CD34	CD44	CD90

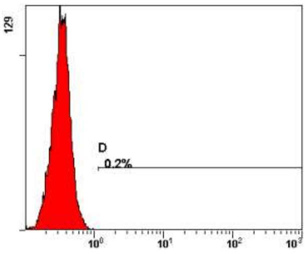

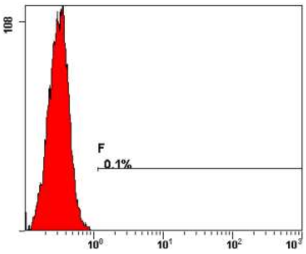

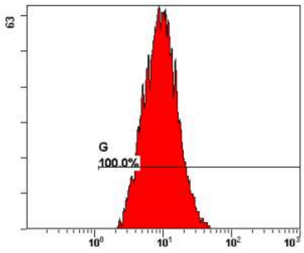

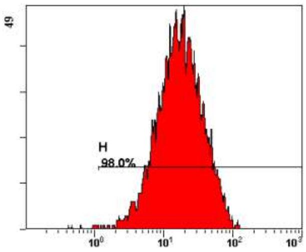

Tibia BMSCs:

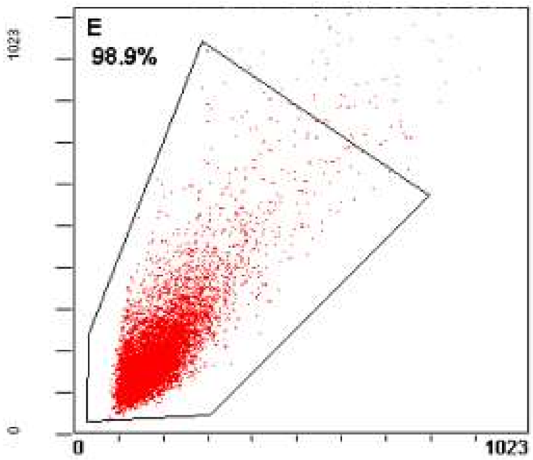

Region	Number	%Total	%Gated	X-Mean	Y-Mean
ALL	10144	100.00	100.00	185	197
E	10031	98.89	98.89	179	188

CD45	CD34	CD44	CD90

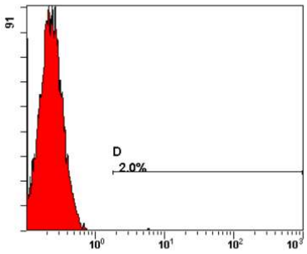

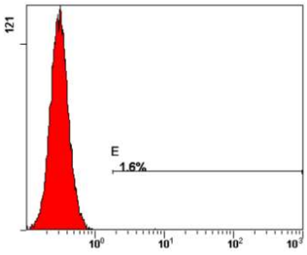

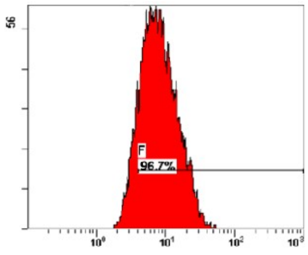

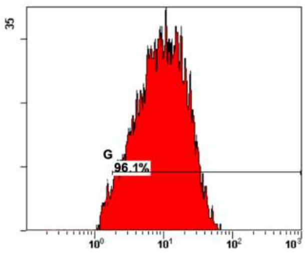

Supplement: Supplementary Data [file srep36129-s1.pdf]
